# Purifying selection of the lysosomal enzymes arylsulfatase A and beta-galactocerebrosidase and their evolutionary impact on myelin integrity

**DOI:** 10.1016/j.jlr.2025.100769

**Published:** 2025-03-05

**Authors:** Matthew A. Luetzen, Richik Chakraborty, Oscar Andrés Moreno-Ramos, Olga Yaneth Echeverri-Peña, Yoko Satta, Adriana M. Montaño

**Affiliations:** 1 Department of Biochemistry and Molecular Biology, School of Medicine, Saint Louis University, St. Louis, MO, USA; 2 School of Medicine, Saint Louis University, St. Louis, MO, USA; 3 Clinical Trials Office, Georgetown Lombardi Comprehensive Cancer Center, Washington D.C., USA; 4 Department of Pediatrics, School of Medicine, Saint Louis University, St Louis, MO, USA; 5 Facultad de Ciencias, Departamento de Ciencias Biológicas, Centro de Investigaciones Genéticas en Enfermedades Humanas (CIGEN), Universidad de los Andes, Bogotá, Colombia; 6 Instituto de Errores Innatos del Metabolismo. Pontificia Universidad Javeriana, Bogotá, Colombia; 7 Department of Evolutionary Studies of Biosystems, SOKENDAI (The Graduate University for Advanced Studies), Hayama, Kanagawa, Japan

**Keywords:** myelin, lysosomal storage disorders, ARSA, GALC, neurodegeneration

## Abstract

The myelin is responsible for providing stability to the axons of the nerve cells, but above all, to improve transmission speed of the nerve impulse in vertebrates. Over 70% of the myelin sheath is composed of lipids and the remaining portion by approximately 2,000 proteins. The myelin sheath has been constantly evolving, and it is known that unusually high concentrations of galactosylceramide (GalCer) and its sulfated form play a major role in the biophysical properties of the myelin. To gain insights of the evolutionary role of GalCer, we have studied two lysosomal enzymes involved in GalCer degradation, arylsulfatase A (ARSA) and galactocerebrosidase (GALC). Deficiency of ARSA or GALC causes demyelinating disorders. We conducted phylogenetic analyses of 105 ARSA and 110 GALC orthologs representing more than 600 million years ago of evolution. We examined *i*) low values of the ratio of nonsynonymous to synonymous nucleotide-substitution rates (dN/dS) indicating purifying selection and *ii*) negative selection of amino acids located in the active site preventing pathogenic mutations. Gene structure analyses showed evidence of rearrangement with gain and loss of exons while there were conserved regions mainly located around the active site. We also found a limited number of sites under positive selection pressure that do not cause alterations to the overall protein structure. Our results indicate that ARSA and GALC have been highly conserved during the evolutionary process to maintain the metabolism of GalCer, which is essential for the integrity of the white matter in vertebrate species.

The nervous system (NS) in vertebrates is accountable for the response to external stimuli, through the transmission of nervous impulses to the central nervous system (CNS). This task is facilitated by the presence of myelin, which is a specialized plasma membrane on the axons of the nerve cells (1). Myelin is formed by the successive winding of the oligodendrocytes’ membrane around neuronal axons in the CNS or Schwann cells in the peripheral nervous system. Its main functions are to provide stability and integrity to the axons and to increase the speed of axonal transmission of nerve impulses through the nodes of Ranvier (2, 3, 4).

Myelin is present in all the vertebrate species (with the exception of jawless basal vertebrates: i.e., lampreys); and it was first acquired by the oldest jawed fish, the placoderms during the Devonian period (4, 5). The conduction velocity in myelinated fibers is proportional to the diameter, whereas in unmyelinated fibers, it is proportional to the square root of the diameter (6). Therefore, there is a clear relationship between the presence of myelin and the effectiveness of nerve impulse’s speed transmission and thus the ability to react to stimuli.

There is a tendency to have slow speed transmission of the nerve impulse with large axon diameter versus fast speed transmission of the nerve impulse with small axon diameter. For example, a giant squid’s axon, with a diameter greater than 200 μm, achieves a transmission speed of no more than 30 m/s, compared with the axons of mammals, which can exceed 100 m/s in fibers with a diameter of only 20 μm due to myelination (5, 7, 8, 9). This feature might be related to the evolution of myelin chemical composition, but it remains to be proven (10). Stiefel *et al.* (8) proposed a new hypothesis stating that during the Mesozoic Marine Revolution, the role of myelin changed from mainly improving the energy efficiency of the action potential conduction to improving conduction speed (11). This hypothesis opens up the possibility that some of the biochemical systems of both synthesis and degradation of the myelin sheath may have evolved prior to determining its functionality.

Lipids represent 70% of the composition of the myelin sheath, including cholesterol, phospholipids, and glycolipids (Fig. 1A–C). At least 2,000 different proteins make the remaining 30% of the total chemical composition (12, 13). Interestingly, myelin exhibits a unique lipid composition with a 2:2:1 ratio for cholesterol:phospholipids:glycolipids (galactosylceramide [GalCer] and sulfatide) (14), which differs to most biological membranes (2.5:6.5:1.0 ratio) (3). Within the lipid part of the myelin sheath, sulfatide represents more than 6%, and it is located in the outer part of the membrane. It has been shown that sulfatide contributes to the long-term stability of myelin structure and to brain development (15, 16, 17, 18). GalCer (or galactocerebroside) is the main glycosphingolipid in brain tissue. In human CNS development, myelination occurs due to oligodendrocyte differentiation and maturation during early embryonic life (19, 20). Prior to myelination, GalCer is absent in the mammalian brain; however, its concentration increases rapidly during myelination (21). GalCer is essential to myelin structure, stability, and function, and it is involved in oligodendrocyte differentiation (3). GalCer is the precursor of 3′-sulfo-galactosylceramide (sulfatide). GalCer and sulfatide become detectable at E17 and predominate at maturity in the adult mouse brain (22).

**Fig. 1:**
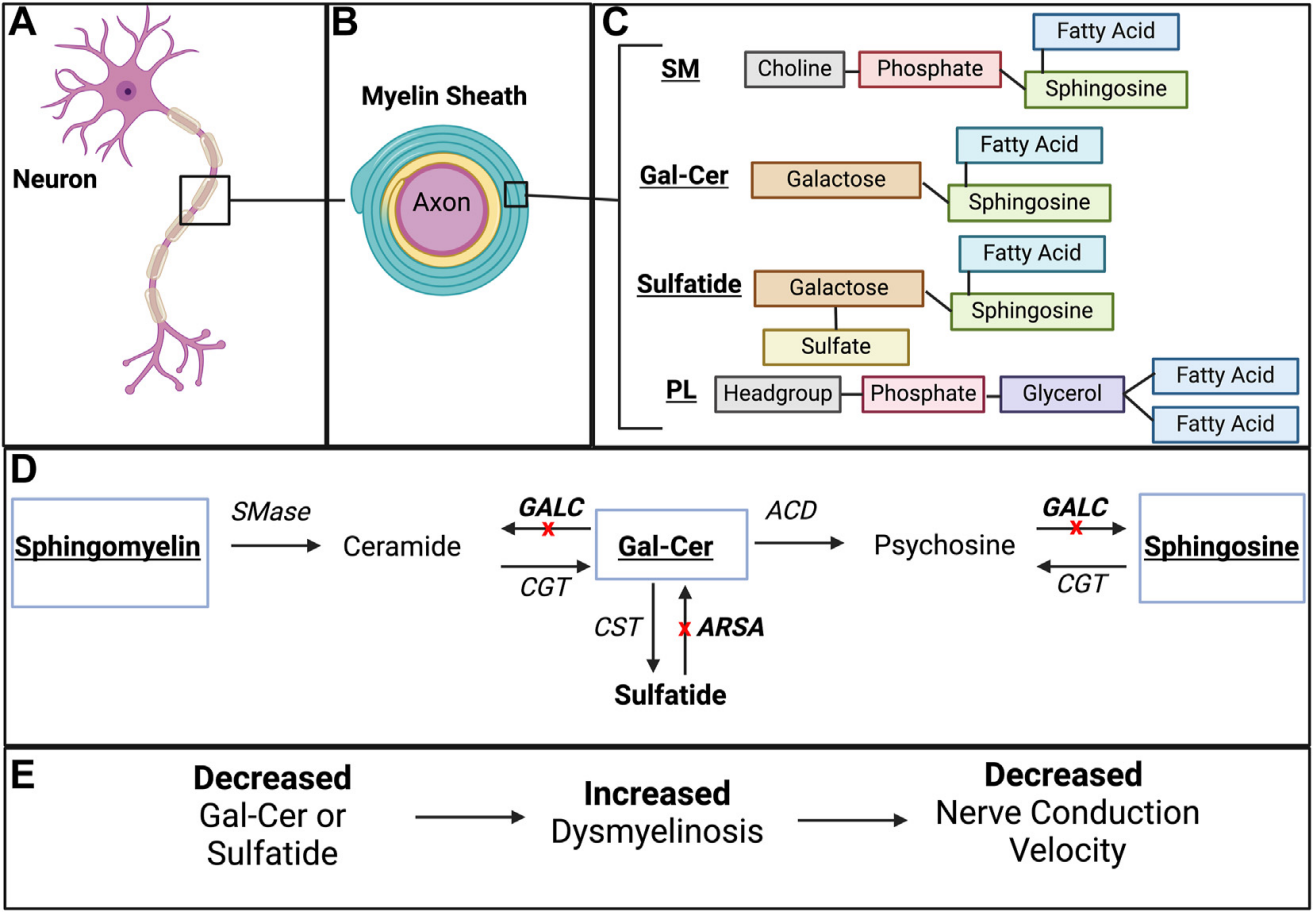
Lipids in the myelin sheath. A, B: The myelin sheath is a lipid-dense insulating layer that enhances action potential propagation and is essential for neurological function. Structurally, the myelin sheath is a concentric modified plasma membrane that tightly wraps around the axon of a neuron. C: At a molecular level, the major lipids of the membrane are cholesterol, phospholipids (PLs), sphingomyelin (SM) and glycolipids, of which GalCer is one of the most abundant. D: ARSA deficiency, also known as MLD, leads to the buildup of sulfatides due to the inability to degrade them into GalCer. GALC deficiency, otherwise known as Krabbe disease, leads to the toxic buildup of psychosine. The accumulation of psychosine and sulfatides due to ARSA or GALC deficiency is toxic to myelin-producing cells and results in demyelination. E: Abnormal decrease of Gal-Cer or sulfatide leads to dysmyelinosis and decreased nerve conduction velocity. ACD, acid ceramidase; CGT, ceramide galactosyltransferase; CST, galactosylceramide sulfotransferase; SMase, sphingomyelinase. Created with BioRender.com.

Degradation of sphingolipids is a necessary part of maintaining lipid homeostasis. The hydrolysis of the sulfate group in position 3 of sulfatide occurs by the action of the lysosomal enzyme arylsulfatase A (ARSA; Enzyme Commission no.: 3.6.1.8) in the presence of its activator Saposin B. Galactosylsphingosine (psychosine) is a metabolic intermediate of GalCer, and together with GalCer, are synthesized by the action of UDP-galactose:ceramide galactosyltransferase on sphingosine in the oligodendrocytes during the production of myelin (23). It has been reported that galactosylsphingosine inhibits cytokinesis, and under normal conditions, its concentration is very low in tissues (24). Galactosylsphingosine levels are kept very low by the action of the enzyme beta-galactosylceramidase (beta-galactocerebrosidase, GALC) in the presence of its activator Saposin A (25, 26) (Fig. 1D). Defects in the catabolism of sphingolipids (i.e., GalCer and sulfatide) cause detrimental effects in the myelin sheath structure triggering a functional deficit of the impulse transmission in a nervous fiber. The deficiencies of ARSA or GALC result in the occurrence of metachromatic leukodystrophy (MLD) or Krabbe disease, respectively. Both clinical phenotypes are characterized by progressive neurodegeneration that involves the CNS and peripheral nervous system and result in vegetative state and early death (26, 27, 28, 29, 30, 31) (Fig. 1E).

There are several evolutionary changes in the myelin sheath, which may affect transmission speed (32, 33, 34). One of them could be related to the homeostasis of sulfatide, which is located in the outer part of the myelin sheath. Changes in the myelin sheath relate to the transmission speed or conformational changes. From an evolutionary viewpoint, changes leading to the generation of specialized nerve fibers in primates should be reflected in the occurrence of selection pressures on the structure of enzymes involved in the synthesis and degradation of the sulfatide. Moreover, the selection pressure should occur at important moments in the evolutionary process where the myelin sheath changed in order to maintain smaller axon diameter, while increasing the speed of transmission of nerve impulse (i.e., habitat change from water to land, etc) (Fig. 1E). Bioinformatics and computational tools have been widely used for the study of molecular evolution and the phylogenetic relationships of proteins associated with a different number of metabolic processes (35, 36, 37, 38, 39, 40). However, there are very few works focused on the phylogenetic analysis of proteins related to myelin sheath metabolism. Modification by evolutionary pressure of ARSA and GALC in differently myelinated species will provide information on adaptation of the myelin sheath and its catabolism to increase speed of transmission without an increase in diameter of the fiber.

This work is aimed to search for selective pressures in the evolution of the *ARSA* and *GALC* genes. Our study postulates the hypothesis that changes in the myelin sheath through evolution were related to selection pressures that occurred over the enzymes responsible for carrying out the degradation of the sulfatide. To evaluate the evolutionary changes and the possible selective pressures that might have occurred, we used public nucleotide and amino acid sequences from the phylum Porifera to primates that represent more than 600 MYA (million years ago) of vertebrate evolution. This is the first report of the evolutionary phylogeny of two enzymes responsible for the first two steps in lipid degradation pathway of the myelin sheath, in the phylum Chordata.

## Materials and Methods

### Identification of ARSA and GALC sequences

ARSA and GALC nucleotides and amino acid sequences were obtained from public databases: National Center for Biotechnology Information and Ensembl (41). Throughout the process to avoid alignment errors and problems associated with incomplete sequences, we only used species for which complete data of amino acid and nucleotide sequences were available. Initially, a BLAST was performed using the human ARSA sequence reported by Ensembl (ENSP00000216124). One hundred five ARSA sequences were chosen, after a strict selection using the ClustalW Multiple alignment application on BioEdit program (42) with manual adjustment and according to length and similarity. For GALC, 110 sequences were selected. For both cases, sequence groups represent nearly 600 MYA chordate phylum evolution. Data include *Saccoglossus kowalevskii* (acorn worm—Enteropneusta) and *Amphimedon queenslandica* (Demospongiae) as an outgroup sequence for ARSA. Verified sequencing and evolutionary availability limited outgroup selection to *Latimeria chalumnae* (*Coelacanth—Sarcopterygii*) for GALC.

### Phylogenetic reconstruction

The final selected alignment was converted to MEGA language (43). Three different approaches were used to estimate evolutionary history of gene reconstruction: Neighbor-Joining algorithm method described by Saitou and Nei (44); close-neighbor-interchange algorithm to find the minimum evolution trees, and algorithms for maximum likelihood approximation (45). For all cases, the standard error was calculated with 1,000 replicates of bootstrap.

### Distance evaluation

The number of nucleotide substitutions measured evolutionary distance among sequences by different methods. *i*) The p-distance was calculated by dividing the number of nucleotide differences by the total number of nucleotides compared, *ii*) according to Jukes and Cantor model, the divergence of nucleotide substitution was estimated, and *iii*) the Tajima-Nei approximation that takes into account unequal rates of substitution among different nucleotide pairs (46, 47, 48). To evaluate the difference among the values obtained by the different methods used, we executed principal component analysis, which is a mathematical procedure that uses an orthogonal transformation to convert a set of observations of possibly correlated variables into a set of values of linearly uncorrelated variables called principal components.

### Nucleotide site divergence and ancestral reconstruction

Several analyses of divergence ratio at nonsynonymous and synonymous sites using available tools in Datamonkey® were conducted (49). To estimate which codon sites were under diversifying positive or negative selection, four different codon-based maximum likelihood methods were evaluated. Initially, the single likelihood ancestor counting was estimated. Afterward, the fixed effect likelihood, the random effects likelihood, and the last internal fixed effects likelihood were evaluated (50, 51). Our criteria to determine a site under positive selection required a consensus of at least two of the methods used at 0.05 significance level.

### Three-dimensional structure prediction

To assess the impact of the sites under possible positive selection pressure on the sequence of the protein, we made the three-dimensional structure prediction in species with different amino acid residues in the specific site under possible positive selection. The three-dimensional protein models were produced on a Dell OptiPlex 3080, Windows 10 Enterprise OS 19042.1288, with Maestro release version 2021-3 from Schrödinger. Protein Data Bank (PDB) ID 1E2S was used for ARSA structure, and PDB ID 3ZR6 was used for GALC (not determined). Default settings were used for preparation of both proteins. In order to make the predictions of different species structure for both enzymes, we used as template the crystal structure reported for human ARSA and murine GALC (52, 53).

## Results

### ARSA and GALC are highly conserved in eukaryotes

**Fig. 2:**
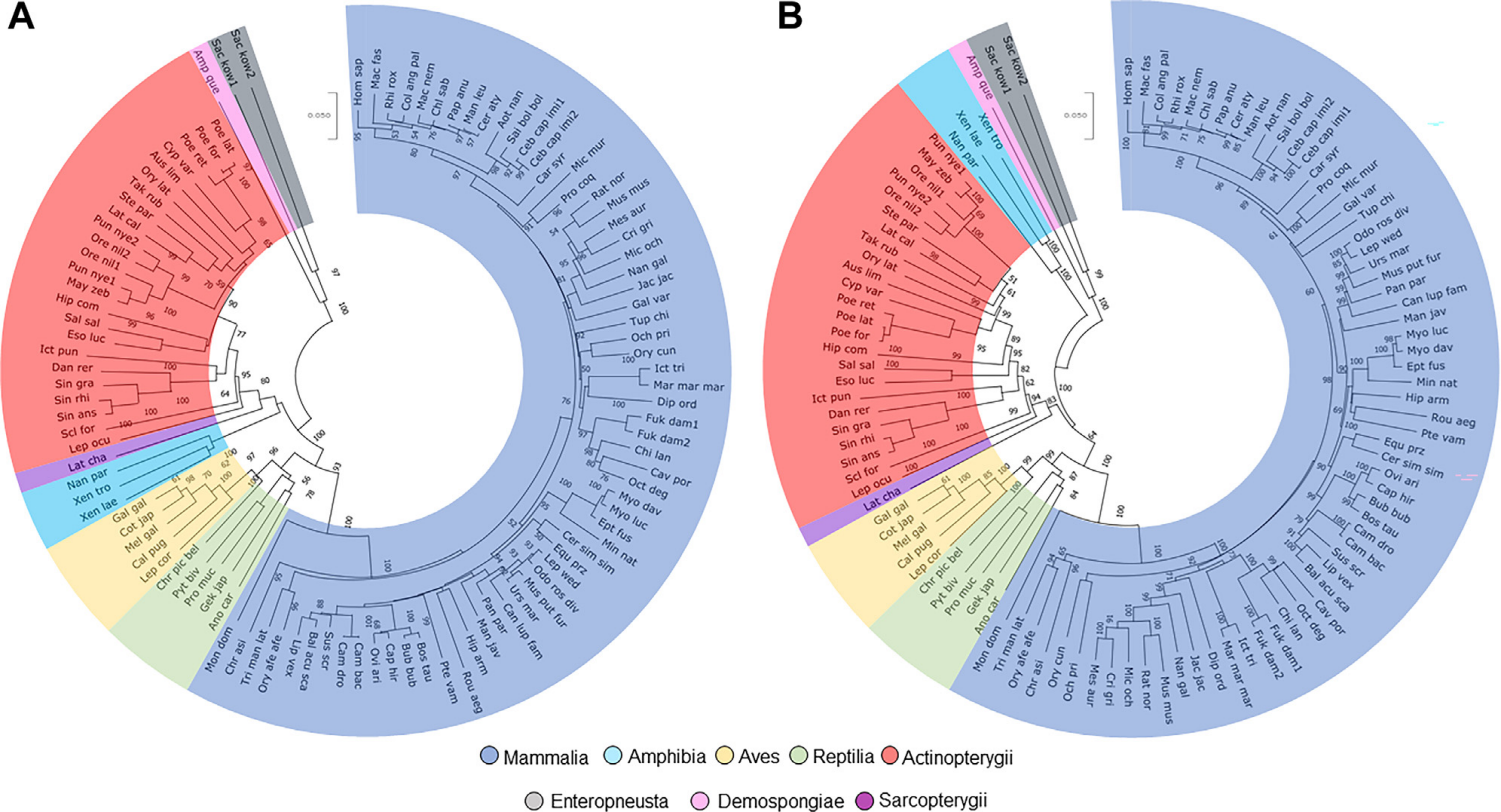
ARSA phylogeny reconstructions using MEGA X. A: The amino acid evolutionary history was inferred using the Neighbor-Joining method. The percentage of replicate trees in which the associated taxa clustered together in the bootstrap test (1,000 replicates) are shown next to the branches. The tree is drawn to scale, with branch lengths in the same units as those of the evolutionary distances used to infer the phylogenetic tree. The evolutionary distances were computed using the p-distance method and are in the units of the number of amino acid differences per site. This analysis involved 105 amino acid sequences. All ambiguous positions were removed for each sequence pair (pairwise deletion). There were a total of 1,007 positions in the final dataset. B: The nucleic evolutionary history was inferred using the Neighbor-Joining method. The percentage of replicate trees in which the associated taxa clustered together in the bootstrap test (1,000 replicates) is shown next to the branches. The tree is drawn to scale, with branch lengths in the same units as those of the evolutionary distances used to infer the phylogenetic tree. The evolutionary distances were computed using the p-distance method and are in the units of the number of base differences per site. This analysis involved 105 nucleotide sequences. Codon positions included were first + second + third + noncoding. All ambiguous positions were removed for each sequence pair (pairwise deletion). There were a total of 3,006 positions in the final dataset. Abbreviations are defined in supplemental Table S1.

**Fig. 3:**
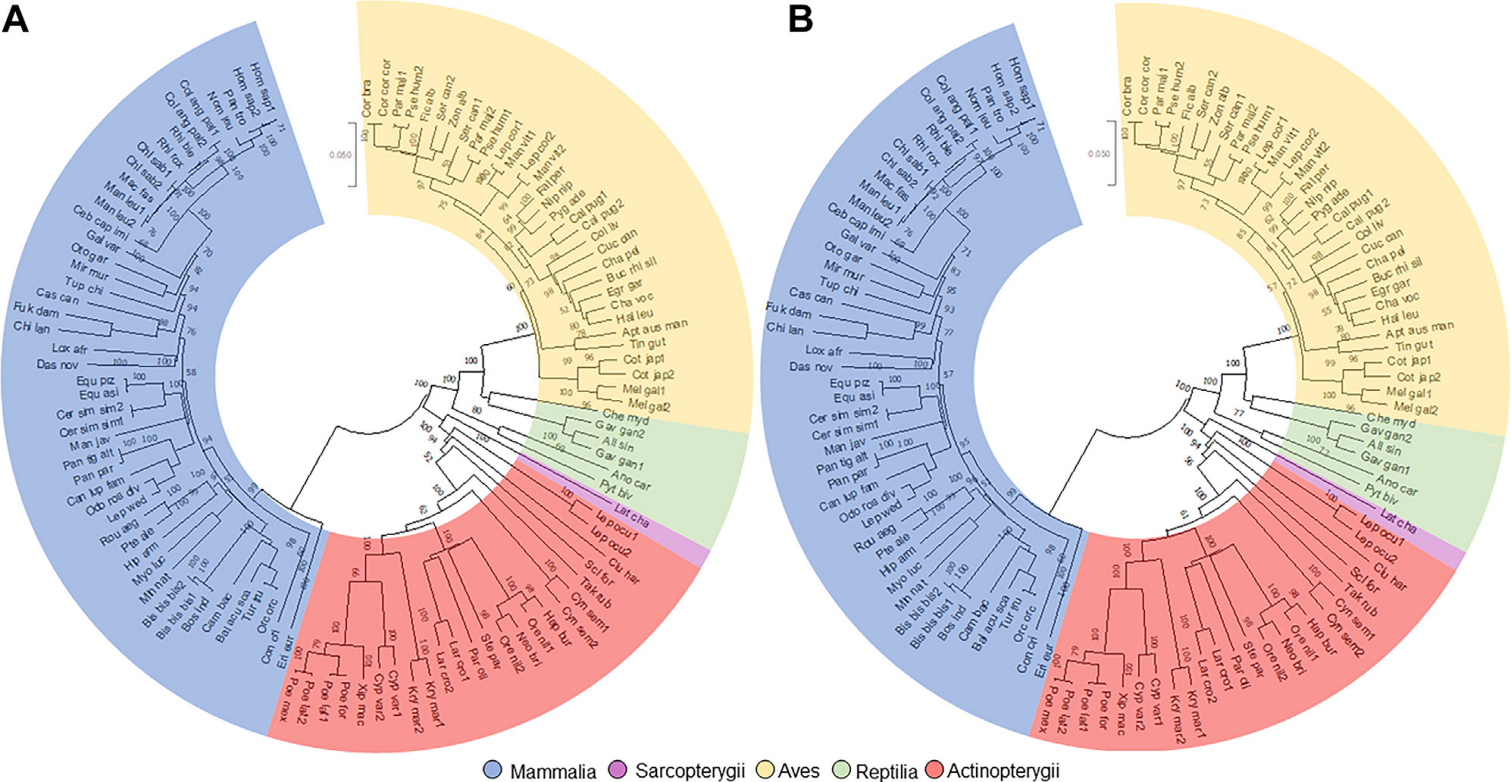
GALC phylogeny reconstructions using MEGA X. A: The amino acid evolutionary history was inferred using the Neighbor-Joining method. The tree is drawn to scale, with branch lengths in the same units as those of the evolutionary distances used to infer the phylogenetic tree. The evolutionary distances were computed using the p-distance method and are in the units of the number of base differences per site. This analysis involved 110 nucleotide sequences. Codon positions included were first + second + third + noncoding. All ambiguous positions were removed for each sequence pair (pairwise deletion option). There were a total of 2,475 positions in the final dataset. B: The nucleic acid evolutionary history was inferred using the Neighbor-Joining method. The percentage of replicate trees in which the associated taxa clustered together in the bootstrap test (1,000 replicates) is shown next to the branches. The tree is drawn to scale, with branch lengths in the same units as those of the evolutionary distances used to infer the phylogenetic tree. The evolutionary distances were computed using the p-distance method and are in the units of the number of amino acid differences per site. This analysis involved 110 amino acid sequences. All positions containing gaps and missing data were eliminated (complete deletion option). There were a total of 1,827 positions in the final dataset. Abbreviations are defined in supplemental Table S2.

To examine the phylogenetic path of ARSA and GALC genes, we identified 105 ARSA (supplemental Table S1) and 110 GALC orthologs (supplemental Table S2) that were retrieved from the National Center for Biotechnology Information database, from species in eight (ARSA) or five (GALC) major taxonomic groups. Alignments were carried out using nucleotide and amino acid sequences. The phylogeny built by three methods, neighbor joining, maximum likelihood, and minimum evolution, showed trees with very similar topology in both ARSA and GALC sequences. The phylogenetic reconstruction of the amino acid and nucleotide sequences of ARSA (Fig. 2) and GALC (Fig. 3) using the Neighbor-Joining method, revealed four main clusters: *i*) mammals including primates and rodents, *ii*) fish, *iii*) aves and reptiles, and *iv*) ancestral species. For ARSA’s phylogenetic analysis, we used *Saccoglossus kowalevskii* (acorn worm—Enteropneusta) and *Amphimedon queenslandica* (sponge—Demospongiae) as outgroup members since they diverged earlier than other organisms analyzed. While sponges do not have an NS, ARSA is present likely for the metabolism of sulfated polysaccharides in their extracellular matrix (54). For GALC, we used *Latimeria chalumnae* (Coelacanth—Sarcopterygii) as an outgroup since it was the most ancient species in the analysis of this gene. The phylogenetic trees for ARSA and GALC have a well-defined topology that follows the eukaryotic evolution.

### ARSA and GALC genes evolved under purifying selection

**Fig. 4:**
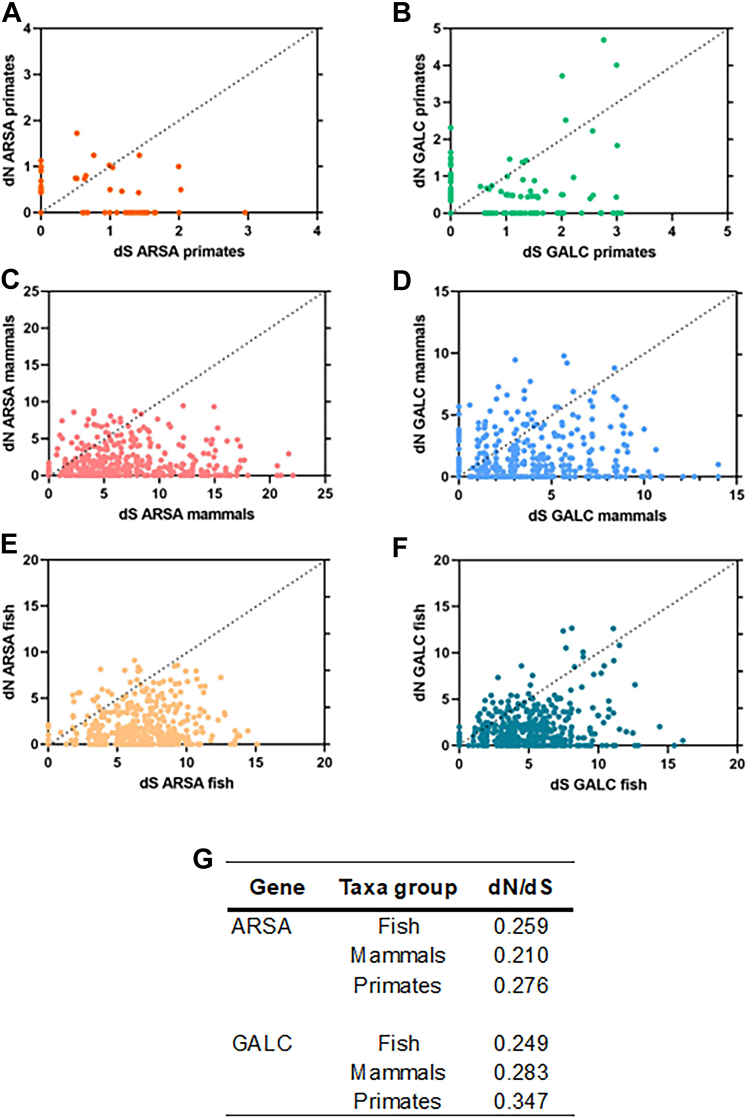
Comparison of synonymous (dS) and nonsynonymous (dN) substitutions in ARSA and GALC. dS and dN substitution rates inferred from 1,007 sites in ARSA and 2,475 sites in GALC. A: ARSA primates, (B) GALC primates, (C) ARSA mammals, (D) GALC mammals, (E) ARSA fish, (F) GALC fish, and (G) ratio of dN/dS for ARSA and GALC.

To investigate the pattern of variation of synonymous (dS) and nonsynonymous (dN) substitution rates in both ARSA and GALC, we explored primates, mammals, and fish sequences. For ARSA gene, comparison of synonymous and nonsynonymous sites in primates (7 species), mammals (62 species), and fish (26 species) showed that there is a lower content of nonsynonymous substitutions compared with synonymous substitutions, which is reflective of purifying selection. In addition, primates showed lower dN and dS compared with mammals and fish species (Fig. 4A, C, E). In summary, the results in different ARSA taxa groups indicate that the ratio is very conserved (dN/dS 0.21–0.27) and constant throughout evolution consistent with purifying selection. For the GALC gene, comparison of synonymous and nonsynonymous sites in primates (14 species), mammals (40 species), and fish (25 species) showed similar trends observed for ARSA gene with conserved dN/dS ratio in mammals (0.28) and fish (0.24). However, there is a higher acceleration rate for nonsynonymous substitutions in primates (dN/dS 0.34) (Fig. 4B, D, F).

### Selective pressures, protein structures, and mutation effects

**Fig. 5:**
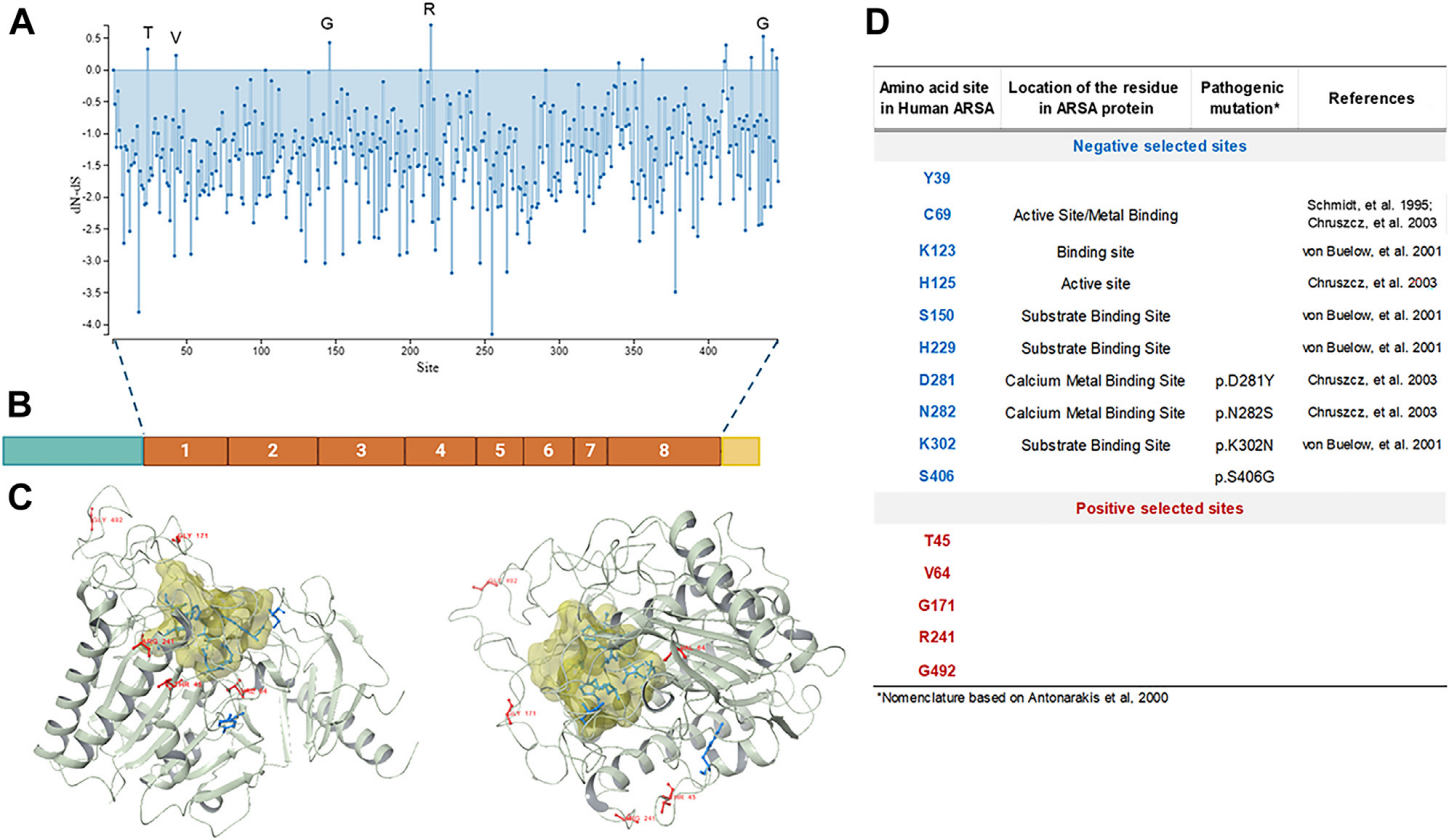
Positive- and negative-selected sites in ARSA. A: ARSA single-likelihood ancestor counting analysis site graph utilizing Datamonkey for analysis of amino acid codon changes under positive selection pressure in 105 sequences with a significance of *P* < 0.05. Single-likelihood ancestor counting uses a combination of maximum-likelihood (ML) and counting approaches to infer nonsynonymous (dN) and synonymous (dS) substitution rates on a per-site basis for a given coding alignment and corresponding phylogeny. ARSA positively selected sequence sites 45, 64, 171, 241, 492 are represented in the figure in red (T: threonine, V: valine, G: glycine, R: arginine, and G: glycine). B: Schematics of human ARSA gene structure. C: Prediction of three-dimensional structure of *Homo sapiens* ARSA. The reconstruction was performed using Schrodinger Maestro 12.9 molecular visualization with Protein Data Bank ID 51289. The positive selection sites, or nonconserved sites, are represented by red coloring of the residues. The negative selection sites, or conserved sites, are represented by the blue coloring of the residues. The active site is represented by the globular tan-colored structure. D: List of positively and negatively selected sites in ARSA with the location of the amino acid residue within the protein and the reported pathogenic variants.

To further understand possible signatures of selection, individual codon sites from ARSA and GALC alignments were evaluated for selective pressure by *i*) nonsynonymous substitutions based on amino acid sequences throughout evolution and *ii*) algorithm consensus provided by Datamonkey (*P* < 0.05). Although there was evidence of purifying selection in ARSA gene, five positive selection sites were predicted in ARSA for codons 45, 64, 171, 241, and 492 (Fig. 5A–D). In ARSA protein, the site 45 corresponds to a nonpolar leucine in fish, alanine in aves and reptiles, changing to a polar threonine in mammals. Site 64 corresponds to a polar serine in aves, reptiles, amphibian, and some fish, which changes to a nonpolar valine in mammals. Site 171 corresponds to threonine in aves and amphibians, and it is promiscuous with several substitutions in reptiles, fish, and mammals. Site 241 corresponds to arginine and glutamine in aves, reptiles, amphibians, and mammals. Last, site 492 has a hydrophilic characteristic in aves, reptiles, amphibians, and mammals. However, this site is highly variable in fish. Overall, none of the predicted positive selection sites are related to a natural variant that causes a pathogenic outcome in humans (Fig. 5D). Further, single likelihood ancestor counting revealed 10 sites to be negatively selected (39, 69, 123, 125, 150, 229, 281, 282, 302, and 406). Sites 69 and125 are located on the active site of ARSA (55, 56), and residues 123, 150, 229, 281, 282, and 302 are located on the binding site of ARSA (57) (Fig. 5C, D). Site 69 is crucial for the activity of the ARSA protein since it is post-translationally modified into *C*_α_-formylglycine, and any mutation in this site will prevent it from becoming active (53, 58). There have been reports of mutations on those highly conserved residues that cause MLD, including D281Y, N282S, K302N, and S406G (59, 60, 61) (Fig. 5D).

**Fig. 6:**
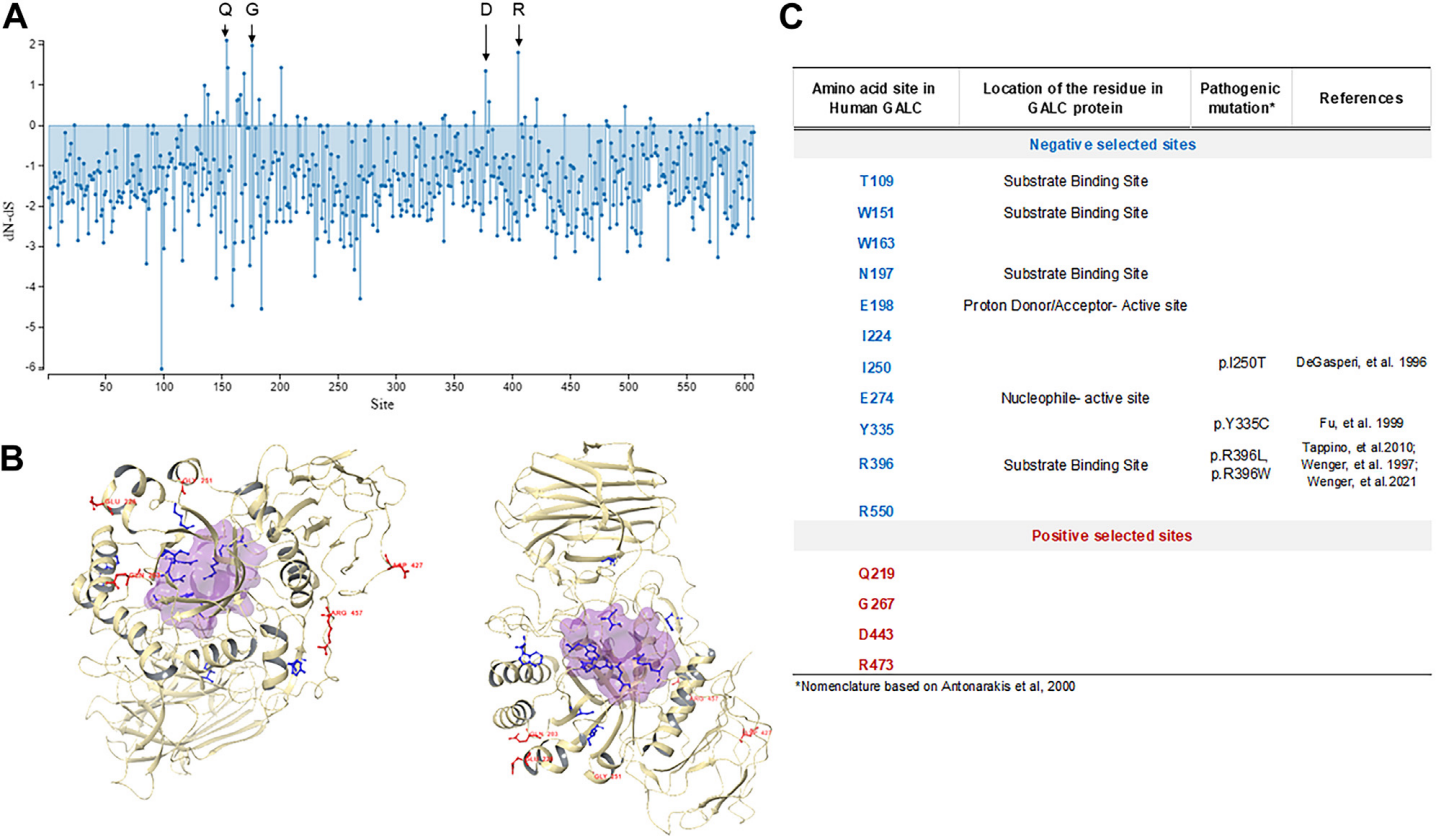
Positive- and negative-selected sites in GALC. A: GALC single-likelihood ancestor counting analysis site graph utilizing Datamonkey for analysis of amino acid codon changes under positive selection pressure in 109 sequences with a significance of *P* < 0.05. Single likelihood ancestor counting uses a combination of maximum-likelihood (ML) and counting approaches to infer nonsynonymous (dN) and synonymous (dS) substitution rates on a per-site basis for a given coding alignment and corresponding phylogeny. GALC positively selected sequence sites 219, 267, 443, and 473 are represented in the figure in red (Q: glutamine, G: glycine, D: aspartate, and R: arginine). B: Prediction of three-dimensional structure for GALC in *Homo sapiens*. The reconstruction was performed using Schrodinger Maestro 12.9 molecular visualization with Protein Data Bank ID 54803 for modeling. The positive selection sites, or nonconserved sites, are represented by red coloring of the residues. The negative selection sites, or conserved sites, are represented by blue coloring of the residues. The active site is represented by the purple globular-colored structure. C: List of positively and negatively selected sites in GALC with the location of the amino acid residue within the protein and the reported pathogenic variants.

Analyses predicted four positively selected sites (219, 267, 443, and 473) in GALC protein (*P* < 0.05) (Fig. 6A–C). Codon 219 corresponds to a very promiscuous site with both hydrophobic and hydrophilic residues among the species. Site 267, 443, and 473 are highly variable with site 443 as aspartate in mammals. None of the positively selected sites had any pathogenic effect in humans. On the other hand, 11 sites were found to be negatively selected. Four of them (109, 151, 197, and 396) play a role in the interaction with the substrate. Site 198 and 274 are located on the active site of GALC protein. Mutations on sites 250, 335, and 396 are known to cause Krabbe disease in humans (62, 63, 64, 65, 66).

In general, sites that were found under positive selection pressure for ARSA and GALC do not result in alterations of the overall structure of proteins. Therefore, there is no detrimental effect in enzyme functionality related to the degradation of the myelin lipids. On the other hand, sites that are highly conserved are essential for the function of the proteins. Any mutation on those residues can cause MLD or Krabbe disease.

### Ancestral reconstruction of ARSA and GALC

**Fig. 7:**
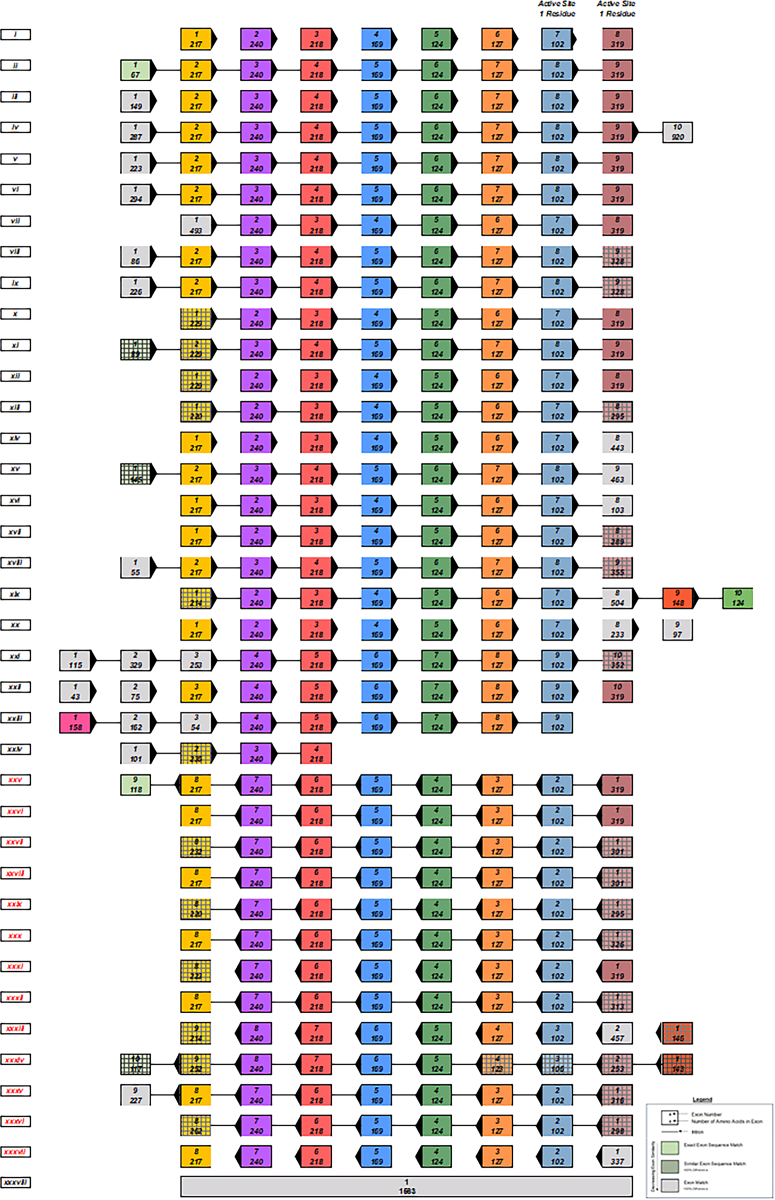
Ancestral exon reconstruction for ARSA displaying exon-intron structure through evolution. Arrows on the exons represent the reading frame direction. Red font represents a reverse in reading frame accompanied by correlating arrow direction. Homologous exons are represented by the same solid vertical color. Gray striping over colored exons within vertical columns represent similar exon sequence of at least 80%. Species were grouped together based on similarity of exon sequences. Different species with less than 5% difference in one exon were grouped. Top number represents the exon in the horizontal sequence of the group. Bottom number represents the number of amino acid residues in the exon. *i*) Col_ang_pal, Cer_aty, Mic_och, Oct_deg, Can_lup_fam, and Equ_prz; *ii*) Car_syr, Lep_wed, Odo_ros_div, Min_nat, Myo_luc, Xen_lae, and Lip_vex; *iii*) Pro_coq, Cam_dro, and Mus_put_fur; *iv*) Man_leu; *v*) Sus_scr; *vi*) Fuk_dam1 and Cer_sim_sim; *vii*) Mac_fas; *viii*) Ict_tri; *ix*) Mar_mar_mar; *x*), Cal_pug; *xi*), Chr_pic_bel; *xii*) May_zeb and Eso_luc; *xiii*) Ory_lat, Scl_for, Tak_rub, Lat_cal, Ste_par, and Poe_ret; *xiv*) Mac_nem; *xv*) Cav_por; *xvi*) Pte_vam; *xvii*) Och_pri; *xviii*) Gek_jap; *xix*) Chr_asi; *xx*) Gal_var; *xxi*) Lat_cha; *xxii*) Tup_chi; *xxiii*) Urs_mar, *xxiv*) Ano_car; *xxv*) Cap_hir, Pan_par; *xxvi*) Jac_jac, Mes_aur, Nan_gal, Rat_nor, Myo_dav, and Ory_cun; *xxvii*) Gal_gal, Mel_gal, and Pyt_biv; *xxviii*) Cot_jap and Ict_pun; *xxix*) Cyp_var, Poe_for, Poe_lat, Hip_com, Sin_ans, Sin_rhi, Sin_gra, and Lep_ocu; *xxx*) Aus_lim; *xxxi*) Hom_sap; *xxxii*) Xen_tro; *xxxiii*) Tri_man_lat; *xxxiv*) Bal_acu_sca; *xxxv*) Mon_dom; *xxxvi*) Sal_sal; *xxxvii*) Ceb_cap_imi1; and *xxxviii*) Amp_que. Abbreviations are defined in supplemental Table S1.

**Fig. 8:**
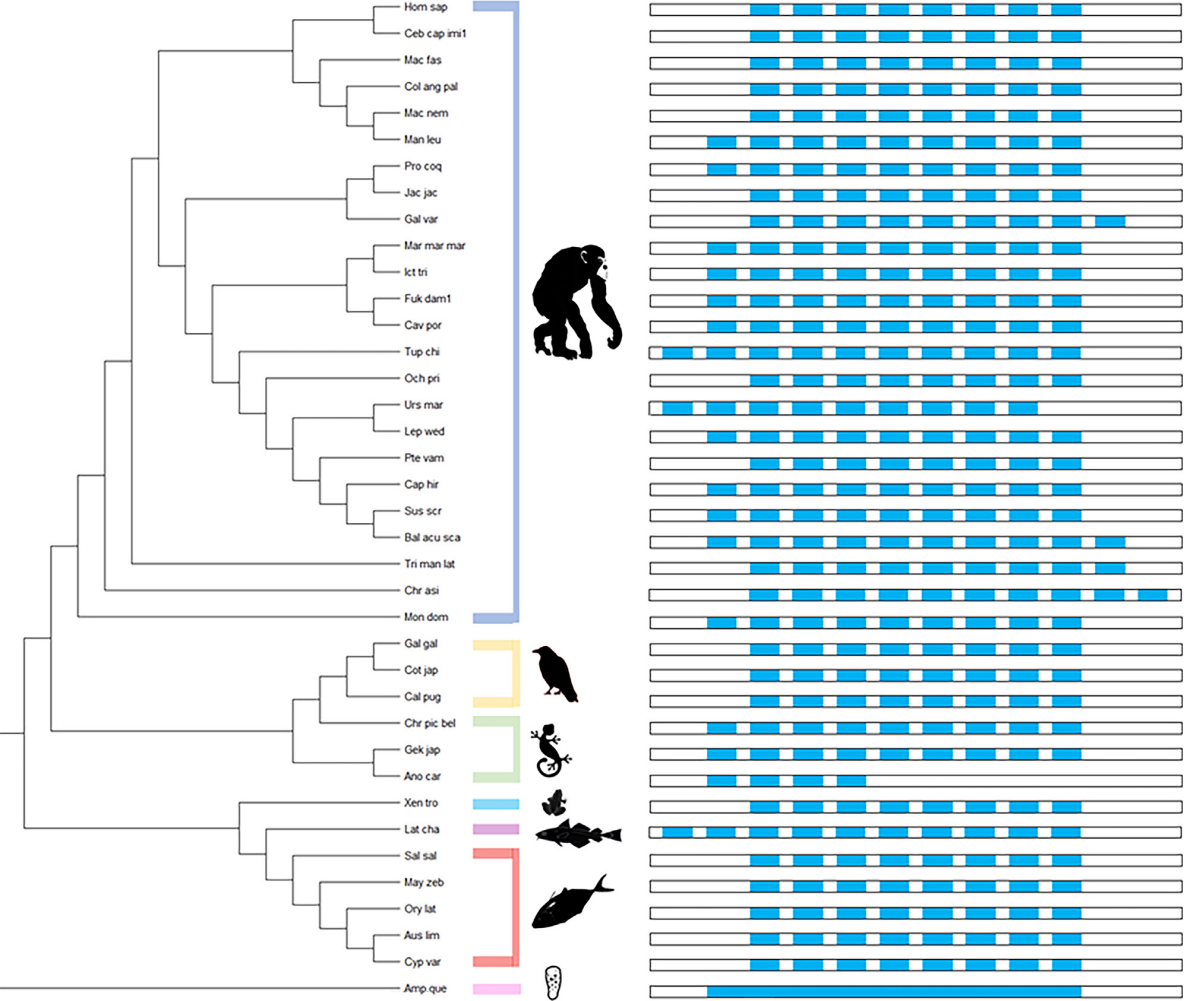
Exon reconstruction timeline of ARSA. One species per group selected for topological representation of p-distances and evolutionary relationship. Species grouped together based on previously characterized classes. Exons are all displayed in 5′ to 3′ forward reading frame. Blue rectangles within the transcript represent exons, and white spaces represent introns. Exons are aligned based on size and similarity.

**Fig. 9:**
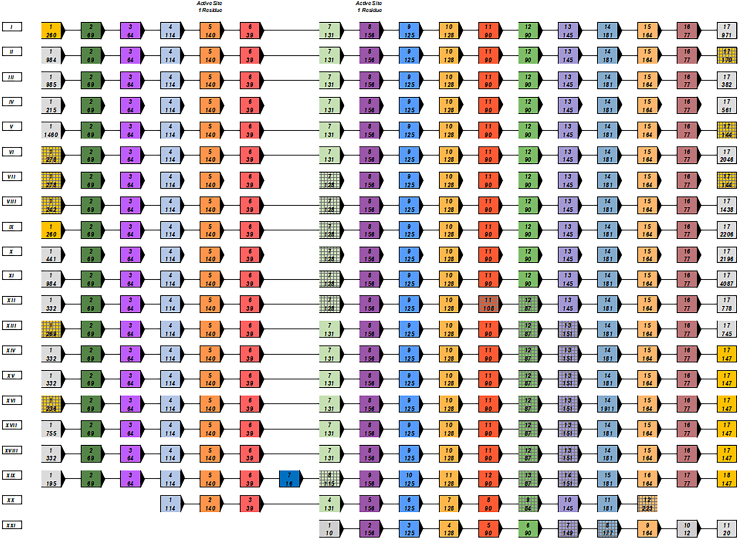
Ancestral exon reconstruction for GALC displaying exon-intron structure through evolution. Arrows on the exons represent the reading frame direction. Homologous exons are represented by the same solid vertical color. Gray striping over colored exons within vertical columns represent similar exon sequence of at least 80%. Species were grouped together based on similarity of exon sequences. Different species with less than 5% difference in one exon were grouped. Top number represents the exon in the horizontal sequence of the group. Bottom number represents the number of amino acid residues in the exon. *i*) Xip_mac, Man_vit1, Lep_cor1, Scl_for, Poe_lat1, Poe_for, Cal_pug1, Cyn_sem1, and Kry_mar1; *ii*) Ore_nil1; *iii*) Lar_cro1; *iv*) Mel_gal1 and Cyp_var1; *v*) Ano_car; *vi*) Poe_mex and Neo_bri; *vii*) Ste_par and Lep_ocu1; *viii*) Ser_can1; *ix*) Zon_alb and Par_maj1; *x*) Lat_cha; *xi*) Cot_jap; *xii*) Clu_har; *xiii*) Chi_lan, Fuk_dam, and Can_lup_fam; *xiv*) Chl_sab2; *xv*) Myo_luc, Oto_gar, Bis_bis_bis1, and Cas_can; *xvi*) Hom_sap, Pan_par, Mir_mur, and Rhi_rox; *xvii*) Man_leu1, Col_ang_pal1, and Nom_leu; *xviii*) Rhi_bie and Pan_tro; *xix*) Pte_ale; *xx*) Fic_alb; and *xxi*) Hap_bur. Abbreviations are defined in supplemental Table S2.

**Fig. 10:**
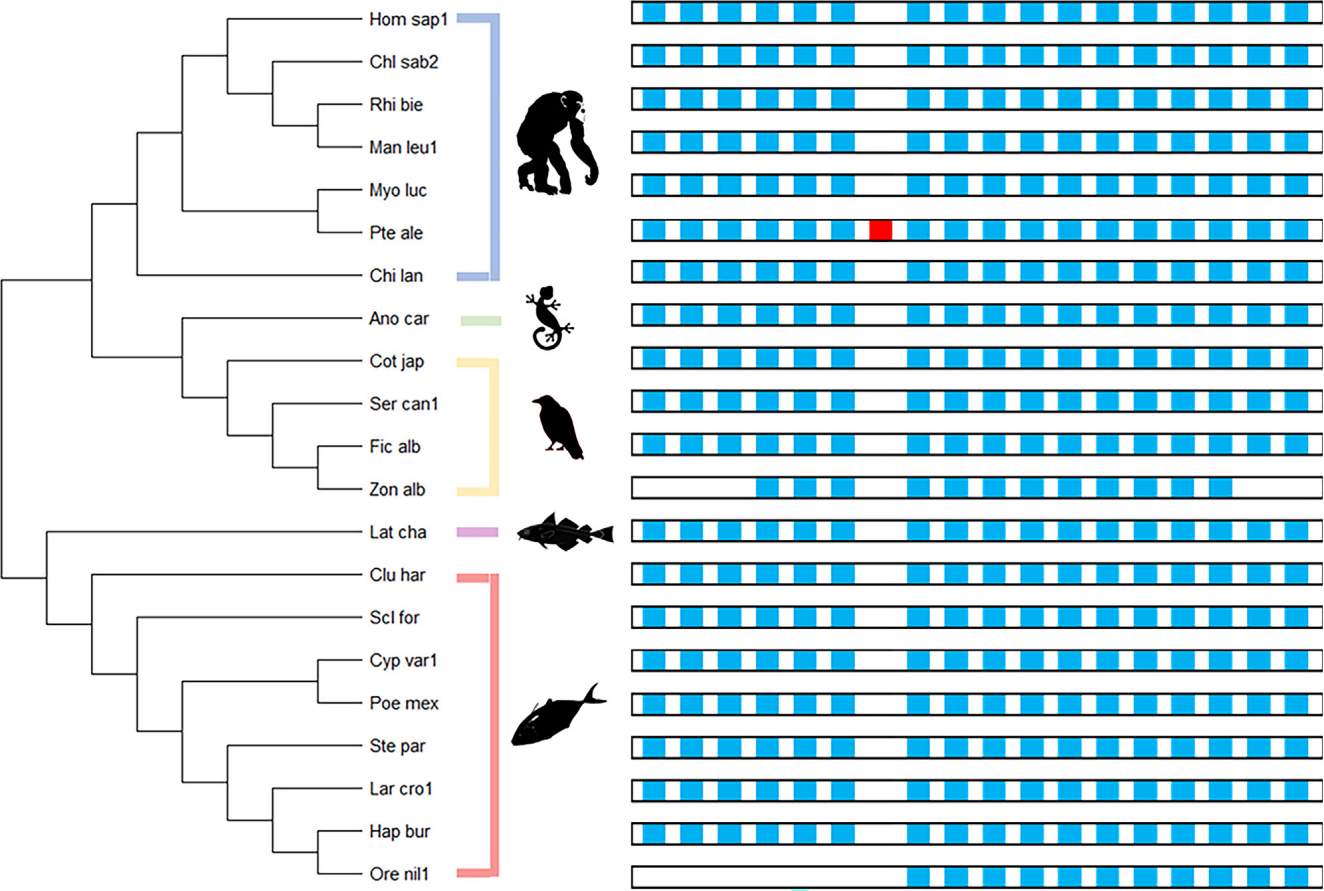
Exon reconstruction timeline of GALC. One species per group selected for topological representation of p-distances and evolutionary relationship. Species grouped together based on previously characterized classes. Exons’ reading frame indicated by forward reading frame (5′ to 3′) and reversal of reading frame (3′ to 5′). Blue rectangles within the transcript represent exons, and white spaces represent introns. Exons are aligned based on size and similarity. The red rectangle indicates a new exon. Ancestral classes were limited to Actinopterygii with lack of GALC presence and transcript characterization in further common ancestors.

To gain insights on the evolutionary history of ARSA and GALC in vertebrates, we reconstructed the history of exon evolution using the amino acid sequence for the exons of ARSA and GALC to reveal the gene structure. We observed different exonic rearrangements among sequences without a clear modification pattern (Fig. 7, Fig. 8, Fig. 9, Fig. 10).

Thirty-eight different types of exon-intron structures were identified among the 105 species. The exons that harbor the catalytic sites C69 and H125 were highly conserved in ARSA throughout evolution as well as eight constitutive exons. We were able to determine an average of 9 to 10 exons for the species that were analyzed with the exception of the green arboreal lizard, which only had four exons providing evidence of exon skipping. We observed that the C- and N-terminal sides of the protein had a tendency of having higher variability, and some exons were fused. ARSA gene inversions were identified in some fish species (order Cypriniformes), Aves (order Galliformes), and some mammals, including a few rodents, a chiroptera, and two primates (capuchin monkeys and humans) (Fig. 7). Exon reconstruction timeline of ARSA revealed that Demospongiae (∼500 MYA) did not have introns. From Actinopterygii, eight core exonic sequences were conserved throughout evolution until *Homo sapiens* (416–420 MYA). There was evidence of reading frame reversing, but eight core exonic sequences were conserved amongst a large variety of classes (Fig. 8) (67).

Twenty-one different iterations of exon–intron structures were identified among the 110 GALC orthologs. Exons that hold the catalytic sites E198 and E274 are highly conserved. We were able to determine an average of 17 exons for the species that were analyzed with the exception of the Collared Flycatcher (Passeriformes) and Burton’s Mouthbrooder (Cichliformes) where exon skipping was observed. There was an exon duplication in the Black Fruit Bat (Chiroptera) toward the N-terminal region of the protein (Figs. 9 and 10). Actinopterygii had only 11 exons (∼433 MYA) (68), in contrast with Reptilia (320 MYA), where we can clearly identify six additional exons in the N-terminal region of GALC.

In spite of the rearrangements observed with loss and gain of exons, and sequence shifts, the active site of both enzymes remained preserved in all the species.

## Discussion

In this study, we showed that two genes, ARSA and GALC, have an impact on the development and maintenance of myelin in vertebrates as evidenced by changes in evolutionary pressures observed throughout the evolution from over 600 MYA. We used Demospongiae or Actinopterygii as outgroups to reconstruct phylogeny and computational analysis inferences to evaluate the presence of positive selection (32, 69, 70, 71). We found that there is a lower content of nonsynonymous substitutions compared with synonymous substitutions, which is evidence of purifying selection for both genes. In addition, we found that the ratio of nonsynonymous to synonymous substitutions is highly conserved in both genes with a slight acceleration observed in GALC primates.

Although the core gene sequence in both genes is conserved in more than 100 species, there are positively selected sites that are promiscuous causing polymorphisms that do not cause any predicted pathogenic outcome in humans or significant changes to the three-dimensional structure of the proteins. It is important to note that under extreme environments, some species could have episodes of relaxed purifying selection due to reduced constraints in some branches, which can be tested in the future (72). On the other hand, negatively selected sites are present on the active site and their functional importance is evidenced by the presence of pathogenic mutations on those sites that lead to devastating neurodegenerative diseases (e.g., MLD or Krabbe disease).

We found evidence of exon and intron rearrangement in both genes; however, the regions around the active site were conserved throughout evolution. According to the three-dimensional structure described by Lukatela *et al.*, active ARSA is an homo-octamer composed by a tetramer of dimers (α_2_)_4,_ and the structure is stabilized by hydrophobic interactions among units. Like other human sulfatases, ARSA presents a cysteine residue at position 69, which suffers a post-translational modification to formylglycine located inside a pocket positively charged that serves as ligand to Mg^2+^. The active site of ARSA is located at the C-terminal end of the major β-sheet and is composed mainly of charged amino acids (53, 73). Our studies revealed that site C69 is negatively selected, which supports the crucial role of this gene by maintaining compact and stable myelin sheath needed for energy efficient signal transmission in the NS.

GALC is a lysosomal enzyme in charge of removing the galactose from the GalCer to produce ceramide. The proposed three-dimensional structure of murine GALC shows that the overall molecule comprises three domains: a central triose-phosphate isomerase barrel, a β-sandwich domain, and a lectin domain. The interfaces formed among each of the domains are very large and consequently are buried together with the triose-phosphate isomerase barrel. In addition, a calcium ion is bound to the lectin domain of GALC in a pentagonal bipyramidal configuration (52, 74). In our study, we identified 11 residues that have essential roles as substrate-binding site or active site, which are negatively selected and thus highly conserved, supporting the important role of GALC in the maintenance of healthy and functional myelin sheath.

Our findings confirmed the high level of conservation of these proteins responsible for the catabolism of sulfatide in the myelin sheath during evolution. In addition, results do not reveal strong evolutionary positive selection. We have found that: *i*) the dN/dS ratio values were less than 0.3 for both proteins; *ii*) amino acids that are located in the active site are under negative selection, and any mutation in this region is pathogenic in nature; and *iii*) there was conservation of the physicochemical nature of the amino acid residues under selection pressure.

Through evolution, the number of dS for both enzymes is higher than that found for dN, indicating that enzymes can suffer modifications in their nucleotide sequence. However, these are not reflected in the translated amino acid sequence, which could significantly affect the structure or functionality of the protein (Fig. 4). We can then conclude that neither of the two enzymes involved in myelin sulfatide metabolism have been subjected to strong positive selection pressures that have had impact on its sequence or structure. This situation is explained by the biological importance of the activity of these proteins in the maintenance of fundamental functions in animals, specifically the ability to give an adequate response to any signal or stimulus that it receives. Sulfatides are vital for the compaction, stability, and organization of myelin in the CNS. They interact with myelin-associated proteins to maintain the structural integrity of the myelin sheath (75). Changes in sulfatide metabolism can disrupt this interaction, leading to demyelination or axonal damage (76). In mammals, particularly humans, sulfatides are abundant in the CNS and are critical for the maintenance of myelin (17). Changes in ARSA and GALC enzymes have implications for myelin formation and maintenance (18, 77).

Myelin is a subcellular structure that emerged with the development of the NS. Through evolution, the physical and chemical properties of myelin have adapted in parallel with the increasing specialization of the NS (32, 78). This evolution has resulted in increased transmission velocity, a greater number of myelin wraps around axons, and a reduction in axonal diameter and length. Consequently, the speed of nerve impulse transmission has increased to hundreds of meters per second. In lower animals with unmyelinated axons exceeding 1 mm in diameter, the transmission speed is limited to approximately 10 m/s. In contrast, animals with myelinated axons, such as *Homo sapiens*, achieve transmission speeds ranging from 6 to 100 m/s with axonal diameters between 2 and 20 μm (1, 79, 80). The observed changes in the NS, particularly in myelin, may be attributed to alterations in its synthesis, degradation, or both. Myelin catabolism involves two key mechanisms related to lipid and protein metabolism. However, the relationship between these processes—whether they occur in parallel, sequentially, or simultaneously—remains unclear (81, 82, 83).

As mammals evolved, with increasingly larger and more complex brains, the demand for efficient myelination also increased. GalCer metabolism evolved in parallel, becoming more finely tuned to support higher rates of myelination, especially in the CNS. The increased ratio of GalCer in mammalian myelin correlates with the need for better insulation of axons and faster signal transmission. Variations in GalCer levels across species also reflect adaptations to different environments and NS needs. For example, in colder-climate species, changes in lipid composition (including GalCer) in myelin may reflect adaptations to maintain nerve function in varying temperatures (84, 85). The evolution of myelin itself is a critical adaptation that allowed vertebrates to develop faster and more energy-efficient NS. GalCer played a pivotal role in this evolutionary leap by providing the necessary structural and functional support for myelin. Organisms with better myelination, supported by efficient GalCer metabolism, would have had a competitive advantage, especially in complex environments that demanded rapid responses and higher cognitive functions. Some advantages of myelination include increased conduction speed, high energy efficiency (through saltatory conduction), and development of compact (small diameter) and efficient NS in complex brains.

Finally, understanding the evolutionary changes in GalCer metabolism could inform therapeutic strategies for treating demyelinating diseases like MLD or Krabbe disease (86). Therapies aimed at enhancing GalCer synthesis or improving the function of enzymes involved in its metabolism could help protect and restore myelin.

## Data availability

All data generated or analyzed during this study are included in this article and its supplemental data. All data in this article are publicly available. The accession numbers of the complementary DNA and amino acid sequences are located in supplemental Tables S1 and S2.

## Supplemental data

This article contains supplemental data.

## Conflict of interest

The authors declare that they have no conflicts of interest with the contents of this article.
