# Supplementary material for: Purifying selection of the lysosomal enzymes arylsulfatase A and beta-galactocerebrosidase and their evolutionary impact on myelin integrity
Source: J Lipid Res. 2025 Mar 5;66(4):100769. doi: 10.1016/j.jlr.2025.100769 (PMC12008523; doi:10.1016/j.jlr.2025.100769)
Supplement: Supplementary material [file mmc1.docx]

## Supplementary information

**Purifying Selection of the Lysosomal Enzymes Arylsulfatase A and Beta-Galactocerebrosidase and their Evolutionary Impact on Myelin Integrity**

Matthew A. Luetzen, Richik Chakraborty, Oscar Andrés Moreno-Ramos, Olga Yaneth Echeverri-Peña, Yoko Satta, Adriana M. Montaño

Corresponding author: Adriana M. Montaño, Ph.D.

Email: [adriana.montano@health.slu.edu](mailto:adriana.montano@health.slu.edu)

This file contains:

Supplementary Tables S1-S2

**Table S1:** Species used for phylogenetic analysis of ARSA and respective GenBank accession numbers.

**Table S2:** Species used for phylogenetic analysis of GALC and respective GenBank accession numbers.
